# Inhibition of RNA degradation integrates the metabolic signals induced by osmotic stress into the Arabidopsis circadian system

**DOI:** 10.1093/jxb/erad274

**Published:** 2023-07-15

**Authors:** Putri Prasetyaningrum, Suzanne Litthauer, Franco Vegliani, Martin William Battle, Matthew William Wood, Xinmeng Liu, Cathryn Dickson, Matthew Alan Jones

**Affiliations:** School of Molecular Biosciences, University of Glasgow, Glasgow G12 8QQ, UK; The James Hutton Institute, Invergowrie, Dundee DD2 5DA, UK; School of Molecular Biosciences, University of Glasgow, Glasgow G12 8QQ, UK; School of Molecular Biosciences, University of Glasgow, Glasgow G12 8QQ, UK; School of Molecular Biosciences, University of Glasgow, Glasgow G12 8QQ, UK; School of Molecular Biosciences, University of Glasgow, Glasgow G12 8QQ, UK; School of Molecular Biosciences, University of Glasgow, Glasgow G12 8QQ, UK; School of Molecular Biosciences, University of Glasgow, Glasgow G12 8QQ, UK; Cardiff University, UK

**Keywords:** Arabidopsis, circadian, drought, osmotic stress, post-transcriptional, RNA degradation

## Abstract

The circadian clock system acts as an endogenous timing reference that coordinates many metabolic and physiological processes in plants. Previous studies have shown that the application of osmotic stress delays circadian rhythms via 3ʹ-phospho-adenosine 5ʹ-phosphate (PAP), a retrograde signalling metabolite that is produced in response to redox stress within organelles. PAP accumulation leads to the inhibition of exoribonucleases (XRNs), which are responsible for RNA degradation. Interestingly, we are now able to demonstrate that post-transcriptional processing is crucial for the circadian response to osmotic stress. Our data show that osmotic stress increases the stability of specific circadian RNAs, suggesting that RNA metabolism plays a vital role in circadian clock coordination during drought. Inactivation of XRN4 is sufficient to extend circadian rhythms as part of this response, with *PRR7* and *LWD1* identified as transcripts that are post-transcriptionally regulated to delay circadian progression.

## Teaser

Post-transcriptional regulation of *PRR7*and *LWD1*transcripts by the exoribonuclease XRN4 enables the circadian system to respond to osmotic stress.

## Introduction

Drought is one of the primary contributors to the yield gap that exists between theoretical yields and those realized in the field (Gupta *et al*., 2020). However, plants’ responses to drought are complex, and so our understanding of underlying signalling pathways remains limited. One of the initial steps in plants’ perception of drought stress is the induction of oxidative stress in the chloroplast (Chan *et al*., 2016a). These stresses lead to the inactivation of the redox-sensitive enzyme SAL1, resulting in the accumulation of 3ʹ-phospho-adenosine 5ʹ-phosphate (PAP), a retrograde signalling molecule that indicates metabolic stress within the chloroplast (Chan *et al.*, 2016b; Koprivova and Kopriva, 2016; Phua *et al.*, 2018). PAP accumulation alters global patterns of transcription and RNA catabolism by inhibiting the activity of 5ʹ–3ʹ exoribonucleases (XRNs; Gy *et al.*, 2007; Kurihara *et al.*, 2012; Crisp *et al.*, 2018), while PAP also serves as a secondary messenger to promote abscisic acid (ABA) signalling (Pornsiriwong *et al.*, 2017). Higher order Arabidopsis mutants lacking all three Arabidopsis XRNs (XRN2, XRN3, and XRN4; *xrn234*) have improved drought tolerance, similar to mutant lines that constitutively accumulate PAP (Hirsch *et al.*, 2011). However, these higher order *xrn234* mutants are unlikely candidates for crop improvement as such plants grow slowly and have a delayed flowering phenotype (Hirsch *et al.*, 2011). In comparison, loss of *XRN4* has modest effects on plant growth and physiology, although roles in seed germination and impaired responses to hormones including ethylene, ABA, and auxin have been reported (Basbouss-Serhal *et al.*, 2017; Wawer *et al.*, 2018; Windels and Bucher, 2018). Instead, XRN4 appears to have a greater role in mediating plants’ responses to abiotic factors including heat and salt stress by contributing to cytosolic RNA degradation (Merret *et al.*, 2013, 2015; Nguyen *et al.*, 2015; Kawa *et al.*, 2020).

Mature mRNAs are protected from degradation by a 5ʹ 7-methylguanosine cap and the 3ʹ polyadenosine tail, with polyadenylation serving as the primary determinant of the degradation rate (Decker and Parker, 1993; Sieburth and Vincent, 2018). Beyond these initial regulatory steps, cytosolic RNA degradation can occur in either a 3ʹ→5ʹ or a 5ʹ→3ʹ direction. The exosome and SUPPRESSOR OF VARICOSE (SOV) contribute to 3ʹ→5ʹ degradation, whereas XRN4 is the primary player in 5ʹ→3ʹ degradation since XRN2 and XRN3 are exclusively localized to the nucleus (Nagarajan *et al.*, 2013; Sieburth and Vincent, 2018). These pathways are broadly conserved across metazoans and fungi, although animals and fungi utilize a functionally equivalent XRN4 orthologue (XRN1) for cytosolic 5ʹ→3ʹ degradation (Kastenmayer and Green, 2000; Nagarajan *et al.*, 2013). Degradation of cytoplasmic RNA via these pathways prevents the generation of siRNAs (Zhang *et al*., 2015; Sieburth and Vincent, 2018), although the function of these partially degraded intermediates remains otherwise unstudied. Instead, recent reports demonstrate unanticipated relationships between these conserved RNA degradation pathways and other aspects of RNA metabolism and processing in yeast (Blasco-Moreno *et al*., 2019; Haimovich *et al*., 2013; Sun *et al*., 2013). In plants, at least a portion of XRN4-mediated degradation occurs co-translationally, leading to *xrn4* seedlings having impaired translation of specific transcripts (Carpentier *et al*., 2020).

We have previously reported that the accumulation of PAP leads to a delay in the circadian system, and that comparable phenotypes are observed in *xrn234* seedlings (Litthauer *et al.*, 2018). The circadian system is a molecular timekeeping mechanism that enables time-of-day to be integrated into a plant’s responses to environmental signals (Millar, 2016). Timing information provided by the circadian system allows anticipation of regular environmental changes (such as dawn and dusk) whilst also modulating gene expression in response to stresses (Greenham and McClung, 2015; Grundy *et al.*, 2015; Baek *et al.*, 2020). Manipulation of the circadian system can improve drought tolerance (Legnaioli *et al*., 2009; Nakamichi *et al*., 2016) and alter water use efficiency (Simon *et al.*, 2020). Due to its potential to improve agronomic traits, the circadian system has been proposed as a key target of breeding programmes (Bendix *et al.*, 2015). We therefore sought to determine how osmotic stress contributes to the regulation of circadian timing.

The circadian system is multifaceted but relies on interlocking transcriptional negative feedback loops that generate daily rhythms of ~24 h (Millar, 2016). Morning-phased components, such as CIRCADIAN CLOCK ASSOCIATED1 (CCA1), work in combination with PSEUDO RESPONSE REGULATOR9 (PRR9), PRR7, and PRR5 to repress gene expression throughout the day (Alabadi *et al.*, 2001; Nakamichi *et al.*, 2010). At night, the Evening Complex [primarily comprising EARLY FLOWERING3 (ELF3), ELF4, and LUX ARRHYTHMO (LUX)] inhibits gene expression (Nusinow *et al.*, 2011; Huang *et al.*, 2016). These waves of repression are complemented by transcriptional activators including LIGHT-REGULATED WD1 (LWD1) and LWD2 that promote expression of morning-phased clock genes (Wang *et al.*, 2011; Wu *et al.*, 2016). Following transcription, proteins such as GIGANTEA contribute to the post-translational regulation of circadian timing (Kim *et al.*, 2007; Cha *et al.*, 2017).

Although primarily examined at the transcriptional level, the contribution of post-transcriptional regulation to the maintenance of circadian rhythms is becoming apparent. Alternative splicing, nuclear export, and nonsense-mediated decay (NMD) all contribute to circadian timing, and the *CCA1* transcript has been reported to be less stable in the presence of light (Yakir *et al*. 2007; Jones *et al.*, 2012; Wang *et al.*, 2012; Macgregor *et al.*, 2013; Kwon *et al.*, 2014; Nolte and Staiger, 2015; Romanowski and Yanovsky, 2015; Mateos *et al.*, 2018; Careno *et al*, 2022). Equally, post-transcriptional regulation is similarly recognized as contributing to a plant’s responses to abiotic stress (Filichkin *et al.*, 2015; James *et al.*, 2018). In this study, we demonstrate that loss of XRN4 activity is sufficient to delay circadian timing, and that osmotic stress limits the degradation of *PRR7* and *LWD1*. Importantly, neither *prr7* nor *lwd1lwd2* seedlings are able to delay their circadian system in response to osmotic stress, demonstrating how signals from environmental stresses can be integrated into the circadian system.

## Materials and methods

### Plant material, growth, and treatments

Plant genotypes used in this work are listed in Supplementary Table S1. Plants were germinated and grown on half-strength Murashige and Skoog (0.5 MS) medium for 5–12 d as described in each figure legend before being transferred to either half-strength 0.5 MS medium or 0.5 MS supplemented with 200 mM mannitol as indicated. Plants were grown under 60 µmol m^–2^ s^–1^ white light in 12 h:12 h light:dark cycles. Relative humidity and temperature were set to 60–70% and 22 °C, respectively.

### Accession numbers

Genes examined in this article can be found in the Arabidopsis Genome Initiative database under the following accession numbers: *APA1*, At1g11910; *APX3*, At4g35000; *ATP3*, At2g33040; *CCA1*, At2g46830; *CCR2*, At2g21660; *ELF4*, At2g40080; *ELF5A-2*, At1g26630; *GIGANTEA*, At1g22770; *IPP2*, At3g02780; *LHY*, At1g01060; *LWD1*, At1g12910; *LWD2*, At3g26640; *PDTPI*, AT2g21170; *PRR7*, At5g02810; *PRR9*, At2g46790; *SAL1*, At5g63980; *TOC1*, At5g61380; *XRN2*, At5g42540; *XRN3*, At1g75660; *XRN4*, At1g54490.

### Hypocotyl measurements

Seedlings were germinated on 0.5 MS medium and grown under 60 µmol m^–2^ s^–1^ white light in 8 h:16 h light:dark cycles for 3 d prior to transfer to plates containing 200 mM mannitol or a mock-treated control. Hypocotyl length was measured at 7 d after germination using ImageJ (Abramoff et al. 2004).

### Luciferase activity

Seedlings were grown in 12 h:12 h light:dark cycles before being sprayed with 3 mM d-luciferin in 0.01% Triton X-100 prior to being returned to entraining conditions for 24 h. The age of seedlings used in each experiment is described in the respective figure legend. Luciferase imaging was completed under constant light conditions (20 µmol m^–2^ s^–1^ constant blue and 30 µmol m^–2^ s^–1^ constant red light) for 5 d. Images were taken every 2 h with a QImaging Retiga LUMO Monochrome Camera controlled by a MicroManager 1.4 script. Circadian parameters were determined using the website biodare2.ed.ac.uk which employs Fourier fast transform-non-linear least squares to calculate circadian parameters (Moore *et al.*, 2014).

### Chlorophyll fluorescence imaging

Chlorophyll fluorescence parameters were recorded with a Fluorimager imaging system (Technologica) as previously described (Litthauer *et al.*, 2015). Patterns of *F*_q_ʹ*/F*_m_ʹ were fitted to cosine waves using FFT-NLLS (Plautz *et al.*, 1997) to estimate circadian period length and additional circadian parameters. Sample size was chosen to achieve a power of 0.8 in a two-sample *t*=test at α=0.05. Previously collected data were used to estimate σ=0.6.

### Assessment of PAP accumulation

Twelve-day-old seedlings grown on 0.5 MS were transferred to 0.5 MS medium supplemented with either 200 mM mannitol or a mock control. Seedlings were returned to light:dark cycles for 2 d prior to harvesting on day 3 of osmotic stress at 4 h intervals and stored at –80 °C until processing. Plant tissue was ground using a TissueLyser (Qiagen-Retsch) and then incubated in 0.1 M HCl for 15 min. Particulates were precipitated twice by centrifugation and the supernatant was added to CP buffer (620 mM citric acid, 760 mM Na_2_HPO_4_, pH 4). The samples were then derivatized with chloroacetyl-aldehyde at 80 °C for 10 min prior to measurement using an HPLC system (Shimadzu) with a Phenomenex Luna 5 µm C18(2) 100 Å LC 150 × 4.6 mm column. The column was equilibrated with 97% (v/v) Buffer A {5.7mM [CH_3_(CH_2_)_3_]_4_NHSO_4_ and 30.5 mM KH_2_PO_4_, pH 5.8} and 3% (v/v) acetonitrile. After injection, the concentration of acetonitrile rose to 33% (v/v) with a linear gradient across 43 min 20 s; the column was then re-equilibrated with 97% (v/v) Buffer A and 3% (v/v) acetonitrile for 6 min 40 s. The concentration of PAP was measured relative to commercially available standards and is presented relative to seedling dry weight.

### Gene expression analyses

For gene expression, 10–15 seedlings were pooled. RNA isolation was performed using TriZol™ (Sigma) based on the manufacturer’s instructions. Genomic DNA contaminants were removed using RNase-free DNase I (Thermo Scientific™) and cDNA was synthesized using the RevertAid First Strand cDNA Synthesis Kit (Thermo Scientific™) with oligo(dT) or random hexamer as specified in the figure legends. The resultant cDNA was used as template for real-time PCR (primers listed in Supplementary Table S2) using the StepOne™ Real-Time PCR System (Applied Biosystems™). Data were processed using the dC_t_ method, and are presented relative to *APA1*, *APX3*, and *IPP2* which have previously been reported to be stable over circadian time (Nusinow *et al*., 2011).

### Measurement of deadenylated RNA accumulation

Accumulation of deadenylated RNA was assessed as previously described (Nagarajan *et al.*, 2019). Seedlings were pre-incubated in 3 ml of incubation buffer (15 mM sucrose, 1 mM PIPES pH 6.25, 1 mM KCl, 1 mM sodium citrate) with aeration (swirling at 100 rpm) in Petri dishes for 15 min. Transcription was inhibited by adding 3 ml of fresh buffer containing 1 mM cordycepin. Vacuum infiltration was performed for 15 min. Tissue was collected 15 min after vacuum release, snap-frozen in liquid nitrogen, and stored at −80 °C prior to RNA extraction. Deadenylated RNA was extracted by retaining the unbound RNA from total RNA following a standard RNA magnetic bead-based oligo(dT) purification (Qiagen RNeasy Pure mRNA Bead Kit; Nagarajan *et al.*, 2019). cDNA was synthesized using a random hexamer oligo. RNA steady-state accumulation is presented relative to *GAMMA SUBUNIT OF MT ATP SYNTHASE* (*ATP3*), *EUKARYOTIC ELONGATION FACTOR 5A-2* (*ELF5A-2*), and *PLASTID ISOFORM TRIOSE PHOSPHATE ISOMERASE* (*PDTPI*).

### RNA stability assay

RNA stability was assessed as previously described (Sorenson *et al.*, 2018). Seedlings were pre-incubated in 3 ml of incubation buffer (15 mM sucrose, 1 mM PIPES pH 6.25, 1 mM KCl, 1 mM sodium citrate) with aeration (swirling at 100 rpm) in Petri dishes for 15 min. Transcription was inhibited by adding 3 ml of fresh buffer containing 1 mM cordycepin. Vacuum infiltration was performed for 15 min. Tissue was collected 30, 60, 120, and 180 min after vacuum release, snap-frozen in liquid nitrogen, and stored at −80 °C prior to RNA extraction. cDNA was synthesized using a random hexamer oligo. RNA steady-state accumulation is presented relative *ATP3*, *ELF5A-2*, *PDTPI*, and *IPP2*.

## Results

### Circadian responses to osmotic stress occur at transcriptional and post-transcriptional levels

**Fig. 1.**
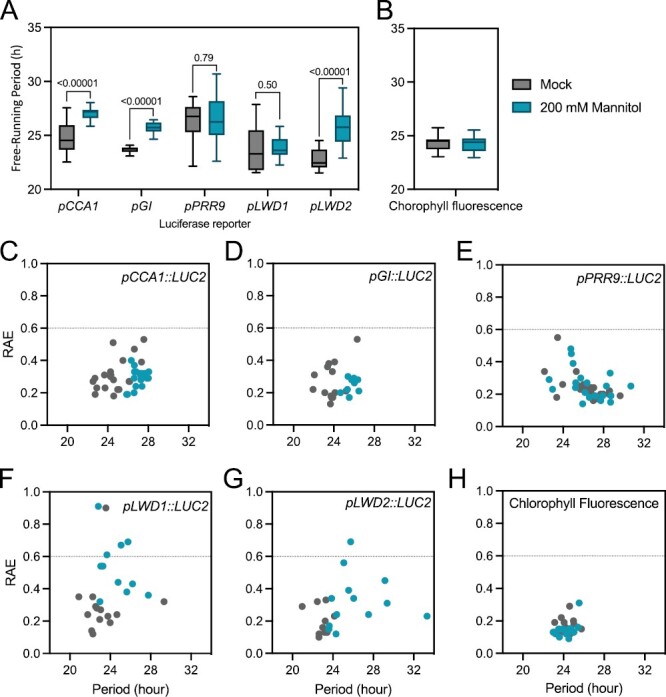
Luciferase reporter constructs highlight varied responses to osmotic stress. (A) Circadian free-running period of *pCCA1::LUC2*, *pGI::LUC2*, *pPRR9::LUC2*, *pLWD1::LUC2*, and *pLWD2::LUC2* reporter constructs in the presence or absence of osmotic stress. Plants were grown on 0.5 MS medium in 12 h:12 h light:dark cycles before transfer to 0.5 MS in the presence or absence of 200 mM mannitol 24 h prior to imaging in constant red and blue light (30 µmol m^–2^ s^–1^ and 20 µmol m^–2^ s^–1^, respectively). A Mann–Whitney multiple *t*-test was used to assess differences in circadian period between treatments. (B) Circadian free-running period of chlorophyll fluorescence in the presence or absence of osmotic stress. Seedlings were treated as described in (A) prior to transfer to constant blue light for imaging. (C–-H) Assessment of rhythmic robustness (relative amplitude error, RAE) against the circadian free-running period for data presented in (A) and (B). An RAE of 0 is indicative of a perfect fit, whereas an RAE of 1 represents the mathematical limits of rhythm detection (Plautz *et al*., 1997).

We were interested in how different aspects of the circadian system responded to osmotic stress. Therefore, we utilized a catalogue of luciferase reporter lines and Chl *a* fluorescence to monitor circadian rhythms following transfer to 200 mM mannitol (Fig. 1; Supplementary Fig. S1). Osmotic stress significantly extends the circadian free-running period (FRP) and reduces bioluminescence when assessed using luciferase reporters driven by the promoter of *CCA1* or *GI*, in line with our initial studies (Fig. 1A, C, D; Litthauer *et al.*, 2018). However, it was noteworthy that *pPRR9::LUC2* seedlings had a much reduced response, whereas *pLWD1::LUC2* reporter lines did not demonstrate an extension of the FRP following osmotic stress (Figs 1A, E, F). Although we were able to determine circadian parameters for *pLWD1::LUC2* and *pLWD2::LUC2* after the transfer to 200 mM mannitol, these oscillations were greatly damped (and presented an increased relative amplitude error; RAE), suggesting that expression of these genes is driven to functional arrhythmia in the presence of osmotic stress (Fig. 1F, G; Supplementary Fig. S1G, I). On the other hand, *pLWD2::LUC2* lines retained an extension of the circadian period following the application of osmotic stress (Fig. 1A, G). In contrast, rhythms of Chl *a* fluorescence were retained in the presence of 200 mM mannitol (Fig. 1B, H). These data demonstrate that only a subset of the circadian system is disrupted following the application of osmotic stress.

### The SAL1/PAP pathway delays the circadian free-running period via cytosolic XRN4

**Fig. 2.**
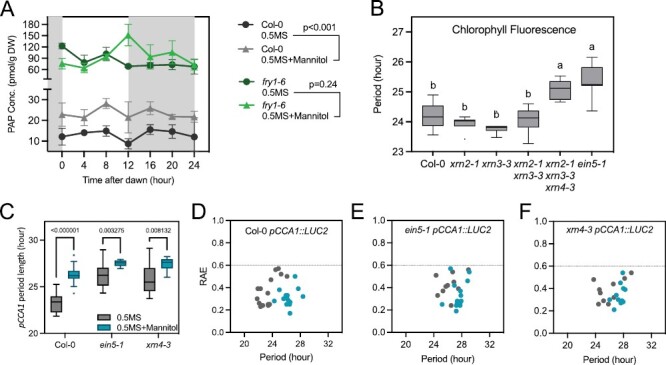
XRN4 contributes towards the extension of the circadian free-running period in response to osmotic stress. (A) Accumulation of PAP in Col-0 and *fry1-6* seedlings. Plants were grown on 0.5 MS medium for 12 d prior to transfer to 200 mM mannitol. Seedlings were maintained in entraining conditions prior to harvest on the third day after application of osmotic stress (48–72 h after transfer). Data are the mean of three biological replicates and analysed using paired *t*-test; the SEM is shown. (B) Circadian free-running period of Col-0 (wild-type), *xrn2-1*, *xrn3-3*, *xrn2-1xrn3-3*, *xrn2-1xrn3-3xrn4-3*, and *ein5-1* seedlings was assessed using chlorophyll fluorescence. Seedlings were grown as described in (A) prior to imaging in the absence of osmotic stress. Data were analysed using one-way ANOVA and Tukey’s multiple comparisons test; the SEM is shown. (C) Circadian free-running period of *xrn4* alleles expressing a *pCCA1::LUC2* reporter construct. Seedlings were grown on 0.5 MS medium in 12 h:12 h light:dark cycles for 5 d before transfer to 0.5 MS in the presence (blue) or absence (grey) of 200 mM mannitol. Data were analysed using two-way ANOVA. (D–F) Assessment of rhythmic robustness (relative amplitude error, RAE) against the circadian free-running period for data presented in (C), with a threshold set to 0.6.

PAP is a retrograde signal that accumulates in response to osmotic stress. Endogenous PAP levels increase as oxidative stress impairs the activity of SAL1, a redox-sensitive phosphatase that would otherwise degrade PAP in the chloroplast and mitochondria (Chan *et al*., 2016a, b). *sal1* seedlings have an extended circadian period (Litthauer *et al.*, 2018), and so we were interested in whether the accumulation of PAP during osmotic stress varied over the course of a day. PAP accumulation is modest in mock-treated wild-type plants, with little variation in PAP levels in Col-0 seedlings during the day (Fig. 2A, *P*=0.268). However, there was a significant increase in PAP accumulation in mannitol-treated wild-type plants compared with a mock-treated control (*P*<0.001). No significant difference in PAP accumulation was observed in plants carrying the *fry1-6* allele of *SAL1* following application of osmotic stress*. sal1* mutants constitutively accumulate PAP, with PAP accumulation substantially higher than that observed in both mock- and mannitol-treated wild-type seedlings (Fig. 2A, *P*=0.24; Litthauer *et al*., 2018). These data suggest that PAP accumulation does not vary over the course of the day, in either the absence or the presence of osmotic stress.

One of the biochemical consequences of PAP accumulation is the inhibition of the XRN family of exoribonucleases, with Arabidopsis expressing three *XRN* orthologues (Nagarajan *et al*., 2013). Previous studies have reported that *xrn234* seedlings exhibit an extended circadian FRP, although they also exhibit a pleiotropic phenotype including impaired growth (Hirsch *et al.*, 2011; Litthauer *et al*., 2018). We were thus interested in determining if specific XRN proteins were sufficient to link the SAL1/PAP signalling pathway into the circadian system. We examined the FRP of the single, double, and triple mutants of three Arabidopsis *XRN* orthologues using Chl *a* fluorescence and luciferase assays. Neither *xrn2-1*, *xrn3-3*, nor *xrn2-1 xrn3-3* seedlings have a significant FRP extension when assessed by chlorophyll fluorescence (Fig. 2B; Supplementary Fig. S2A). However, we observed that the *ein5-1* allele of *xrn4* displayed an impaired circadian system, with a circadian FRP 1 h longer than in wild-type controls, and was indistinguishable from *xrn234* seedlings (Fig. 2B; Supplementary Fig. S2A). A similar extension of the FRP was observed using a *pCCA1::LUC2* reporter construct, with both *ein5-1* and *xrn4-3* alleles of *xrn4* exhibiting an extended FRP compared with the wild type (Fig. 2C; Supplementary Fig. S2C–H, τ=23.19 ± 0.34, 26.34 ± 0.68, and 26.70 ± 0.60 h in wild-type, *ein5-1*, and *xrn4-3* lines, respectively). To further investigate whether the circadian system of *xrn4* seedlings retained a response to osmotic stress, we assessed the response of *ein5-1* and *xrn4-3* seedlings to osmotic stress. Our results suggest that these seedlings continued to demonstrate a modest response to osmotic stress (*P*<0.01), although this response was much less pronounced than in wild-type seedlings (Fig. 2C–F; Supplementary Fig. S2E–H). These data suggest that XRN4 contributes to the extension of the circadian FRP in response to osmotic stress, while also indicating that additional mechanisms contribute to the integration of osmotic stress into the circadian system.

### Osmotic stress limits the degradation rate of *LWD1*, *LWD2*, and *PRR7* transcripts

**Fig. 3.**
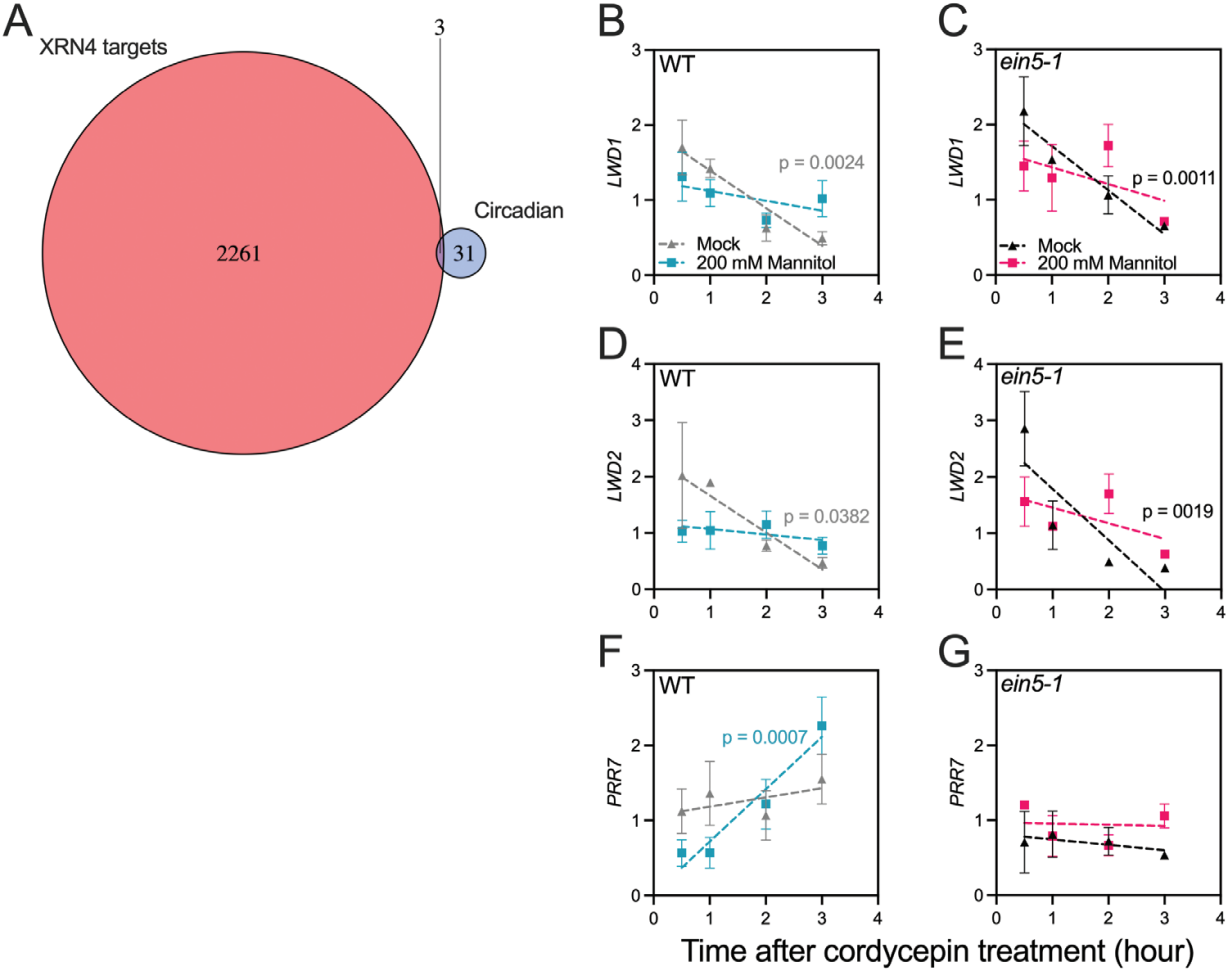
Stability of circadian transcripts is increased by osmotic stress. (A) Comparison of putative XRN4 degradation targets and characterized components of the circadian system. Putative XRN4 targets were previously identified by PARE or GMUCT (Nagarajan *et al*., 2019; Carpentier *et al*., 2020), while defined circadian components were drawn from a previous review (Hsu and Harmer, 2014). (B and C) Assessment of *LWD1* total transcript stability in Col-0 (B) and *ein5-1* (C) seedlings in the presence or absence of osmotic stress. (D and E) Assessment of *LWD2* total transcript stability in Col-0 (D) and *ein5-1* (E) seedlings in the presence or absence of osmotic stress. (F and G) Assessment of *PRR7* total transcript stability in Col-0 (F) and *ein5-1* (G) seedlings in the presence or absence of osmotic stress. Seedlings were grown on 0.5 MS for 6 d prior to transfer to a mock control medium or medium containing 200 mM mannitol. Sampling and application of 0.5 mM cordycepin was completed at ZT4 on day 7, at 28 h after transfer to experimental conditions. Data are reported relative to the average accumulation of *ATP3*, *ELF5A-2*, *PDTPI*, and *IPP2* transcripts. A simple linear regression applied for each combination of genotype and treatment was applied from *t*=0.5; *P*-values are shown when the slope is significantly different from 0. Data are the mean of at least three independent experiments, *n*>10. Error bars indicate the SEM.

Over 5500 genes are misregulated in *xrn4* seedlings (Gregory *et al*., 2008; Merret *et al.*, 2015; Carpentier *et al.*, 2020), and ~2200 Arabidopsis transcripts have been proposed as XRN4 substrates following parallel analysis of RNA ends or genome-wide mapping of uncapped transcripts (PARE and GMUCT, respectively; Fig. 3A; Nagarajan *et al.*, 2019; Carpentier *et al.*, 2020). Of these candidate substrates, only three (*PRR7*, *LWD1*, and *LWD2*) are established components of the circadian system, although *COLD CIRCADIAN RHYTHM2/GLYCINE RICH PROTEIN7* (*CCR2/GRP7*), part of a slave suboscillator, is also a candidate *XRN4* substrate (Fig. 3A; Heintzen *et al*., 1997; Farré *et al*., 2005; Wu *et al*., 2008). In order to validate these RNA-seq data, we assessed the stability of *LWD1*, *LWD2*, and *PRR7* transcripts in the presence or absence of osmotic stress. Experiments were completed in the presence of cordycepin to inhibit transcription (Supplementary Fig. S3A). Degradation of *LWD1* was reduced relative to controls following the application of osmotic stress in both wild-type and *ein5-1* seedlings (Fig. 3B, C). However, the rate of *LWD1* degradation was comparable in both wild-type and *ein5-1* seedlings, suggesting that factors beyond XRN4 contribute to the degradation of this transcript (*P*>0.66, Fig.3A–C). Similarly, the stability of *LWD2* was enhanced by osmotic stress in both genotypes examined, with little difference in degradation rate between wild-type and *ein5-1* seedlings (Fig. 3D, E). In contrast to *LWD1* and *LWD2*, the rate of *PRR7* degradation was comparable with that of the stable control transcripts in mock conditions, suggesting that *PRR7* RNA is relatively stable (Fig. 3F, G). Indeed, *PRR7* RNA appeared more stable than controls following the application of osmotic stress in wild-type plants (Fig. 3F, *P*<0.01). Our data show that osmotic stress limits the degradation rate of *LWD1*, *LWD2*, and *PRR7*, but highlight that additional factors work with XRN4 to regulate degradation of these transcripts.

**Fig. 4.**
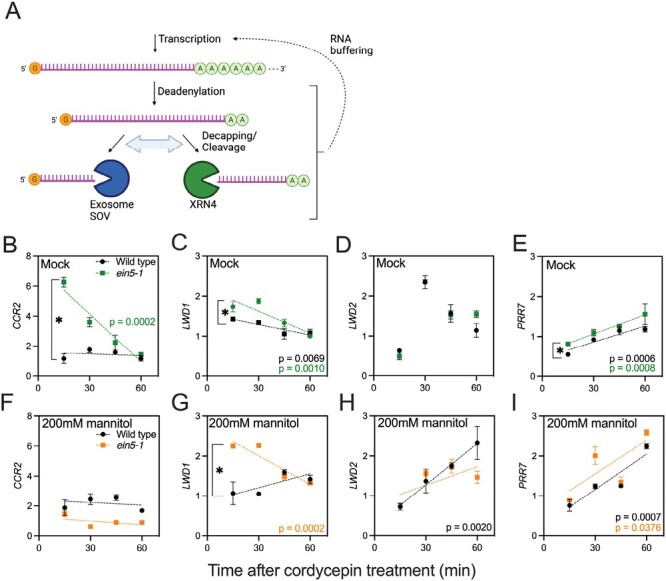
mRNA degradation is modulated by osmotic stress and XRN4 activity. (A) Outline of RNA degradation pathways in Arabidopsis. Following deadenylation, RNAs are degraded in a 3ʹ–5ʹ direction by the exosome or SOV (note that Col-0 is an *sov* mutant; Zhang *et al*., 2010). XRN4 degrades RNA in a 5ʹ–3ʹ direction following endonucleic cleavage or 5ʹ decapping. Compensatory adjustments between these parallel pathways occur following mutation of RNases, and changes in transcription rates in these cases (‘RNA buffering’) have also been reported (Sorenson *et al*., 2018). Created with BioRender.com. (B–E) Assessment of deadenylated transcript accumulation in the wild type and *ein5-1*. *CCR2* (B), *LWD1* (C), *LWD2* (D), and *PRR7* (E) RNAs were monitored. Application of 0.5 mM cordycepin and subsequent sampling was completed at ZT4 on day 7. (F–I) Assessment of deadenylated transcript accumulation in the wild type and *ein5-1* following the application of osmotic stress. *CCR2* (F), *LWD1* (G), *LWD2* (H), and *PRR7* (I) RNAs were monitored. Seedlings were grown on 0.5 MS for 6 d prior to transfer to 200 mM mannitol; cordycepin was added at ZT4, at 28 h after application of osmotic stress. Data are reported relative to the average accumulation of *ATP3*, *ELF5A-2*, and *PDTPI* transcripts. Data are the mean of at least three independent experiments, *n*>10. A simple linear regression for each combination of genotype and treatment was applied from *t*=15; colour-coded *P*-values are shown when the slope is significantly different from 0. Separately, post-hoc Mann–Whitney tests were applied to compare initial deadenylated RNA levels at *t*=15. Data are the mean of at least three independent experiments, *n*>10. Error bars indicate the SEM.

Both transcription and RNA degradation contribute to RNA abundance within cells, with evidence that disruption of cytosolic RNA degradation can influence transcription via ‘RNA buffering’ (Fig. 4A; Sieburth and Vincent, 2018; Sorenson *et al.*, 2018). XRN4 plays a key role in these processes, with roles in both co-translational decay and cytosolic 5ʹ–3ʹ RNA decay (Fig. 4A; Kastenmayer and Green, 2000; Nagarajan *et al.*, 2019; Carpentier *et al.*, 2020). Since XRN4 degrades deadenylated RNAs, we next assessed the accumulation of partially degraded RNA targets (i.e. RNA without an adenylated tail, Fig. 4A) compared with control RNAs that have not been defined as XRN4 targets (Fig. 4; Supplementary Fig. S3; Sorenson *et al*., 2018). Accumulation of deadenylated *CCA1* (which is not a proposed XRN4 target) was consistent across our experiment and did not vary between the wild type and *ein5-1* (Supplementary Fig. S3B). Deadenylated *CCR2* levels were significantly increased in *ein5-1* seedlings compared with wild-type controls (*P*<0.01), with the number of deadenylated transcripts decreasing over time in *ein5-1* seedlings (presumably as a consequence of exosome-mediated degradation, *P*<0.0002, Fig. 4A, B). Deadenylated *LWD1* transcripts were similarly elevated in *ein5-1* seedlings (despite being less pronounced than for *CCR2*, *P*<0.01), although in this case there was no significant difference in degradation rate between the wild type and *ein5-1* (*P*=0.0504, Fig. 4C). In contrast, patterns of deadenylated *LWD2* accumulation were complex, with deadenylated *LWD2* being more stable than control RNAs following the application of cordycepin (Fig. 4D). We did not observe any significant differences in deadenylated *LWD2* accumulation between wild-type or *ein5-1* samples at any time point. Levels of deadenylated *PRR7* were consistently elevated in *ein5-1* seedlings compared with the wild type, although there was no difference in degradation rate between the two lines (*P*<0.01, Fig. 4E). Deadenylated *PRR7* RNAs also increased over time relative to control deadenylated RNAs (*P*<0.001, Fig. 4E). These data suggest that XRN4 contributes to degradation of deadenylated *CCR2*, *PRR7*, and *LWD1*. However, the relative contribution of other degradation pathways (such as the exosome) appears to vary, with deadenylated *LWD2* and deadenylated *PRR7* in particular being more stable than control RNAs.

We next examined how the application of osmotic stress altered the accumulation of deadenylated RNAs (Fig. 4F–I). Interestingly, the accumulation of deadenylated *CCR2* was greatly reduced in *ein5-1* mutants in the presence of 200 mM mannitol compared with the mock control, in agreement with the enhanced accumulation of the full-length transcript during drought stress (Fig. 4F, *P*<0.001; Carpenter *et al*., 1994). In contrast, *ein5-1* seedlings continued to accumulate more deadenylated *LWD1* than wild-type controls (Fig. 4G, *P*<0.01). Application of osmotic stress increased the accumulation of deadenylated *LWD2* in both wild-type and *ein5-1* seedlings, with deadenylated *LWD2* continuing to accumulate throughout cordycepin treatment (Fig. 4H). Although deadenylated *PRR7* continued to accumulate during the experiment, there was no difference in either deadenylated *PRR7* accumulation (*P*=0.06) or degradation rate (*P*=0.897) between wild-type and *ein5-1* samples during osmotic stress (Fig. 4I). Overall, our data demonstrate that osmotic stress affects the accumulation of deadenylated transcripts, but that additional factors beyond XRN4 contribute to this phenotype.

**Fig. 5.**
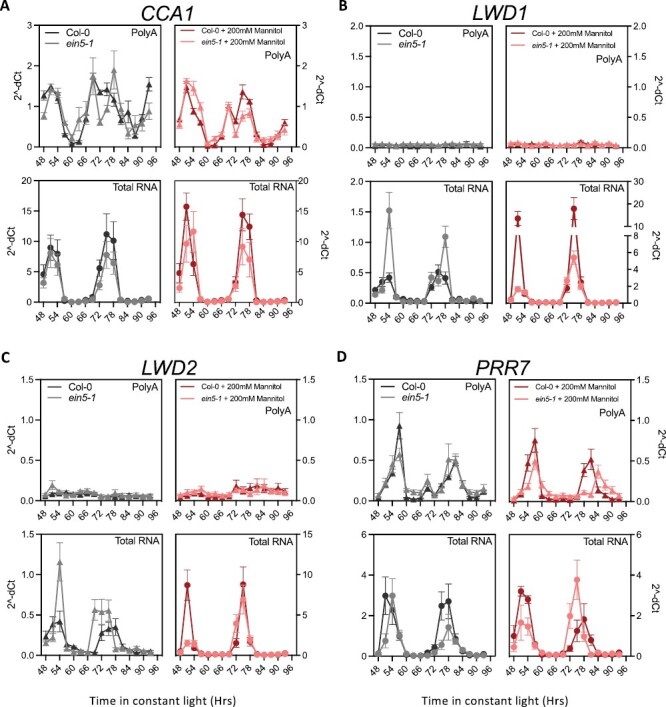
Relative polyadenylated and total RNA abundance of selected circadian clock genes following application of osmotic stress. Wild-type and *ein5-1* seedlings were grown on 0.5 MS medium in 12 h:12 h light:dark cycles for 5 d before transfer to 0.5 MS in the presence (red) or absence (black) of 200 mM mannitol. Seedlings were returned to entraining conditions for 24 h prior to transfer to continuous white light (60 µmol m^–2^ s^-1^). Fold change in *CCA1* (A), *LWD1* (B), *LWD2* (C), and *PRR7* (D) is presented relative to three circadian reference genes given in Supplementary Table S1. cDNA was synthesized using either an oligo(dT) primer or a random hexamer to obtain poly(A)^+^ and total transcript, respectively. Data were normalized using the 2–dC_t_ method. Data are representative of at least three independent experiments (*n*>10). Error bars indicate the SEM.

Since osmotic stress delays circadian progression and alters transcript stability, we were interested in whether the accumulation of polyadenylated and total RNA fractions was altered in stressed seedlings over circadian time. Osmotic stress was applied using 200 mM mannitol, and mRNA was extracted to generate cDNA using oligo(dT) (to assess polyadenylated mRNA) or a random hexamer (to capture RNA decay intermediates in addition to polyadenylated mRNA; Fig. 5; Supplementary Fig. S4). We first examined polyadenylated transcript levels and were interested to note that *CCA1* polyadenylated mRNA accumulation remained robust following application of 200 mM mannitol (Fig. 5A). This contrasts with the general reduction of luciferase bioluminescence observed following osmotic stress (Supplementary Fig. S1). Additionally we found that steady-state levels of *LWD1* or *LWD2* polyadenylated mRNA were extremely low in either the presence or absence of mannitol despite *pLWD1::LUC* and *pLWD2::LUC* displaying circadian rhythms (Figs 1A, F, G, 5B, C; Wang *et al.*, 2011). Furthermore, polyadenylated *PRR7* transcript peak levels were comparable between mock and osmotically stressed seedlings (Fig. 5D).

We next compared the relative accumulation of polyadenylated RNA and total RNA over circadian time (Fig. 5). Interestingly, we observed that patterns of polyadenylated RNA did not always align with total RNA within cells. These differences were particularly prominent for *LWD1* and *LWD2* transcripts, with total RNA of *LWD1* and *LWD2* displaying circadian rhythmicity as previously demonstrated by luciferase reporter lines, whereas polyadenylated *LWD1* and *LWD2* species did not accumulate (Figs 1F, G, 5B, C). The discrepancy between polyadenylated and total *LWD1* and *LWD2* RNA highlights a strong post-transcriptional regulation of these transcripts. Additionally, we observed a dramatic increase in the total mRNA of *LWD1* and *LWD2* in both wild-type seedlings and *ein5-1* seedlings subjected to osmotic stress (Fig. 5B, C). Conversely, these increases in total RNA following osmotic stress were not apparent in *PRR7* or *CCA1* species (Fig. 5A, D), suggesting that *LWD1* and *LWD2* total RNA species are particularly sensitive to osmotic stress. These differences between promoter activity, polyadenylated mRNA accumulation, and total RNA (Figs 1, 5) demonstrate how different aspects of transcript metabolism vary over circadian time.

We were next interested in whether the loss of XRN4 altered the accumulation of total RNA over circadian time scales. Despite the loss of XRN4, the rhythmic pattern of *LWD1* and *LWD2* total RNA was retained in *ein5-1* plants, with a slight increase in peak levels of both *LWD1* and *LWD2* total RNA in *ein5-1* seedlings in mock conditions (Fig. 5B, C). Similar to wild-type seedlings, the application of mannitol stress elevated the accumulation of both *LWD1* and *LWD2* total RNA. However, it was noteworthy that *LWD1* total RNA levels were lower in *ein5-1* seedlings compared with the wild type following the application of osmotic stress. In contrast to *LWD1* and *LWD2* total RNA, only modest differences in *CCA1* and *PRR7* total RNA were observed in *ein5-1* seedlings compared with wild-type controls (Fig. 5A, D). These observations of total RNA accumulation suggest that circadian accumulation of *LWD1* and *LWD2* total RNAs is greatly influenced by XRN4 activity but also indicate a complex effect beyond simply degrading the target transcripts, potentially involving RNA buffering mechanisms (Sieburth and Vincent, 2018).

### *PRR7* and *LWD1* enable the circadian response to osmotic stress

**Fig. 6.**
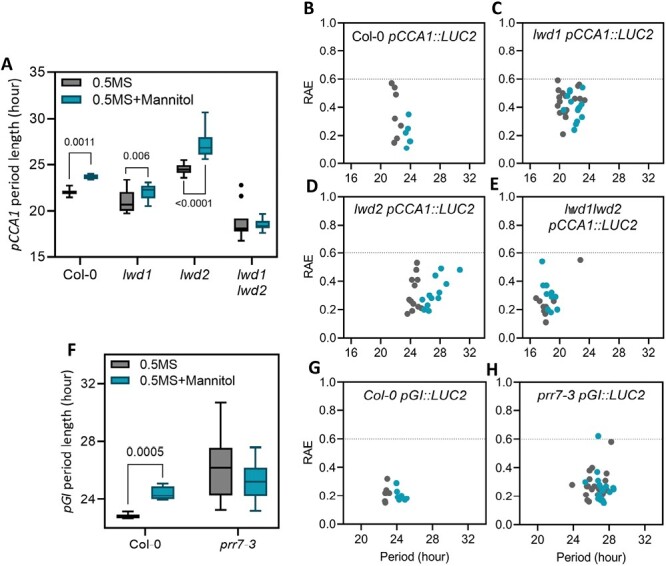
Loss of *LWD1*, *LWD2*, or *PRR7* perturbs circadian responses to osmotic stress. (A) Circadian free-running period of *pCCA1::LUC2* in wild-type, *ein5-1*, *lwd1*, *lwd2*, and *lwd1lwd2* backgrounds. *P*-values for the difference in the circadian free-running period following osmotic stress are shown (Mann–Whitney multiple *t*-test). (B–E) Assessment of rhythmic robustness (relative amplitude error, RAE) against the circadian free-running period for data presented in (A), with a threshold set to 0.6. (F) Circadian free-running period of *pGI::LUC2* reporter lines in wild-type and *prr7-3* backgrounds. *P*-values for the difference in the circadian free-running period following osmotic stress are shown (Mann–Whitney multiple *t*-test). (G and H) Assessment of rhythmic robustness (RAE) against the circadian free-running period for data presented in (F), with a threshold set to 0.6. Plants were grown on 0.5 MS medium for 5 d and transferred to 0.5 MS with or without 200 mM mannitol 24 h before imaging under constant red and blue light (30 µmol m^–2^ s^–1^ and 20 µmol m^–2^ s^–1^, respectively).

Given the role of osmotic stress in the degradation of *LWD1*, *LWD2*, and *PRR7* (Figs 3, 4) we were interested to determine if disruption of these genes was sufficient to alter the plants’ responses to osmotic stress. We first assessed whether *prr7*, *lwd1*, *lwd2, lwd1 lwd2*, and *xrn4* seedlings retained a hypocotyl extension phenotype in response to osmotic stress (Supplementary Fig. S5). All genotypes exhibited significantly shorter hypocotyls following the application of 200 mM mannitol, suggesting that osmotic stress is still experienced by each of these genotypes (*P*<0.001; Šídáks multiple comparisons test, Supplementary Fig. S5). We next assessed if *PRR7*, *LWD1*, or *LWD2* was necessary for the extension of the circadian period observed in response to mannitol treatment (Fig. 6; Supplementary Fig. S6). *lwd1* p*CCA1::LUC2* seedlings have a short period phenotype in the absence of osmotic stress (Airoldi *et al.*, 2019) but retain the extension of the circadian period following the application of mannitol (Fig. 6A–C). *lwd2* p*CCA1::LUC2* seedlings demonstrated a longer FRP than the wild type during both control and osmotic stress treatments, although the lengthening in the FRP in response to osmotic stress remained (Fig. 6A, D). *lwd1 lwd2 pCCA1::LUC2* seedlings displayed a pronounced shortening of the FRP (Wang *et al.*, 2011), but did not display an increase in the FRP in response to osmotic stress (*P*>0.05, Fig. 6A, E). We then investigated the contribution of *PRR7* towards the circadian response to osmotic stress. Interestingly, although robust circadian rhythms were maintained in *prr7-3 pGI::LUC2* seedlings transferred to 200 mM mannitol, we did not observe an extension in the FRP (Fig. 6F–H). These data demonstrate that PRR7 is necessary to maintain the proper response of the circadian system to osmotic stress, and suggest that LWD1 and LWD2 redundantly contribute towards the extension of the FRP as part of this response.

## Discussion

### Post-transcriptional regulation distinguishes the accumulation of polyadenylated mRNA from circadian patterns of promoter activity and RNA decay intermediates

Our initial experiments using luciferase bioluminescence reporters suggested that individual luciferase reporters within the circadian system were differentially regulated in response to osmotic stress, with *LWD1* and *PRR9* promoter-driven lines presenting a diminished circadian response to the application of osmotic stress (Fig. 1). The divergence between the behaviour of different luciferase reporters has previously been reported and may arise in part from tissue-specific expression patterns (Endo *et al*., 2014; Nimmo *et al*., 2020; Hall *et al*., 2002; Haydon *et al*., 2013). Our data suggest that *CCA1*-, *GI*-, and *LWD2*-driven luciferase activity is primarily derived from tissues that respond to osmotic stress, whereas *PRR9-* and *LWD1-*driven rhythms are predominant in less responsive tissue. It will be of great interest to determine whether these differences are reflective of the circadian system adapting to osmotic stress.

Although modern circadian molecular biology is founded upon luciferase reporter constructs that reveal circadian rhythms of reporter activity (Millar *et al.*, 1992), post-transcriptional regulation of some transcripts has been apparent for several years. For example, uniform levels of *LIGHT HARVESTING CHLOROPHYLL BINDING PROTEIN* (LHCB1*3) transcript are maintained despite luciferase activity driven from this promoter being rhythmic (Millar and Kay, 1991; Millar *et al.*, 1992). Conversely, *NITRATE REDUCTASE2* is transcribed constantly, and yet has rhythmic mRNA accumulation (Pilgrim *et al*., 1993). In our study, it is apparent that polyadenylated *LWD1* RNA is arrhythmic in constant light, despite a *pLWD1::LUC* reporter line and *LWD1* total RNA displaying circadian regulation (Figs 1, 5). A similar phenotype is observed for *LWD2* transcripts (Figs 1, 5). The length of the polyadenylated tail correlates negatively with gene expression in Arabidopsis (Parker *et al*., 2020), while removal of the polyadenylation signal is an important initial step in RNA degradation (Fig. 4A; Sieburth and Vincent, 2018). The lack of significant accumulation of polyadenylated *LWD1* and *LWD2* in constant light may therefore indicate that *LWD1* and *LWD2* are rapidly transcribed and degraded, although additional experimentation will be required to test this hypothesis. We were also interested to note that peak accumulation of *PRR7* total RNA preceded the phase of peak polyadenylated *PRR7* RNA by several hours (Fig. 5D). Differences in post-transcriptional processing presumably contribute to the disparities we observe between luciferase reporter activity and steady-state transcript accumulation in response to osmotic stress (Figs 1, 5, 6; Supplementary Figs S1, S5).

### RNA degradation via XRN4 contributes to the maintenance of circadian rhythms

PAP accumulates during osmotic stress, leading to the inhibition of exoribonuclease activity (Fig. 2A; Dichtl *et al.*, 1997; Estavillo *et al.*, 2011; Litthauer *et al.*, 2018). The accumulation of PAP precipitates an extension of the circadian period through the inactivation of XRN exoribonucleases, although it was not apparent from previous studies whether this phenotype was due to increased RNA polymerase II 3ʹ read-through resulting from the nuclear-localized XRN2 and XRN3 or instead arose from catabolism in the cytosol via XRN4 (Crisp *et al.*, 2018; Litthauer *et al.*, 2018; Carpentier *et al.*, 2020). Our studies, along with other recent work in this field, demonstrate that the loss of XRN4 is sufficient to induce extension of the circadian FRP (Fig. 2B–F; Careno *et al.*, 2022). This highlights the contribution of cytosolic RNA degradation to the maintenance of circadian rhythms. Loss of XRN4 was sufficient to induce accumulation of deadenylated *LWD1* and *PRR7* transcripts, although circadian rhythms of *PRR7, LWD1*, and *LWD2* total RNA were still observed in *ein5-1* seedlings (Figs 3–5; Nagarajan *et al.*, 2019). While we cannot exclude a role for XRN4 in the degradation of additional circadian transcripts, further time points harvested throughout the diel cycle will be necessary to obtain a comprehensive dataset describing all clock genes regulated by XRN4, as *XRN4* accumulation is constant in both long-day and short-day conditions (Litthauer *et al.*, 2018). Indeed, it will also be of interest to understand the contribution of XRN4 towards the translation of circadian proteins during osmotic stress, given the contribution of XRN4 to co-translational decay (Merret *et al.*, 2015; Yu *et al*., 2016; Carpentier *et al.*, 2020).

The application of osmotic stress induces changes in both steady-state RNA accumulation and the accumulation of partially degraded RNAs (Figs 3–5). Although the differences in total RNA accumulation may be caused in part by the PAP-mediated inhibition of XRN4 activity, it is apparent that XRN4 is not the sole contributor to RNA stability (Figs 3–5). For example, the unanticipated reduced accumulation of *LWD1* and *LWD2* total RNA at peak times in *ein5-1* seedlings during osmotic stress indicates that additional factors (such as transcriptional regulation or compensatory RNA degradation mechanisms) are also perturbed by the loss of XRN4 activity (Fig. 5; Liu and Chen, 2016). In yeast, the XRN4 functional orthologue XRN1 couples transcription with RNA decay, shuttling into the nucleus as part of a feedback mechanism to regulate mRNA accumulation and translation (Blasco-Moreno *et al*., 2019; Haimovich *et al*., 2013; Sun *et al*., 2013). While this latter mechanism has not been explicitly reported in plants, global analysis of RNA decay in *sov* seedlings reveals communication between cytoplasmic decay and the transcriptional machinery (Sieburth and Vincent, 2018; Sorenson *et al.*, 2018). Since the activity of XRN4 only accounts for a portion of the phenotypes observed, these data support the hypothesis that the circadian system adapts to osmotic stress through post-transcriptional regulation. Further research is necessary to understand which of these changes is directly precipitated by osmotic stress rather than being an indirect consequence of circadian perturbation.

### *PRR7* and *LWD1* contribute to circadian responses to osmotic stress

Although little has been reported regarding the contribution of LWD1 and LWD2 to abiotic stress, PRR7 is increasingly recognized as a crucial circadian component that contributes metabolic information to the molecular timekeeper (Liu *et al.*, 2013; Webb *et al.*, 2019). Since *prr7-3* and *lwd1 lwd2* seedlings demonstrate impaired circadian responses to osmotic stress (Fig. 6; Nagarajan *et al.*, 2019), it is possible that altered accumulation of total *LWD1*, *LWD2*, and *PRR7* transcripts following osmotic stress (Fig. 5) contributes to the observed extension of the circadian FRP. However, the biological function of these RNAs remains to be determined given that changes in polyadenylated RNA remain modest in constant light (Fig. 5).

Previous work has reported enrichment of PRR7 targets that are responsive to abiotic stress (Liu *et al.*, 2013). Indeed, the preponderance of reports linking PRR7 to abiotic stress responses suggests that PRR7 is a central component of plants’ responses to abiotic stresses such as heat, shade, and drought (Liu *et al.*, 2013; Kolmos *et al.*, 2014; Blair *et al.*, 2019; Zhang *et al.*, 2020). A relatively high percentage (28%) of PRR7 targets are also ABA regulated, with more than a third of PRR7 target genes possessing ABA-responsive elements (Liu *et al.*, 2013). It is therefore possible that regulation of *PRR7* by XRN4 provides an additional pathway for PAP to modulate ABA-induced signalling (Pornsiriwong *et al.*, 2017). This idea would also align with the proposed role of PRR7 as a dynamic integrator of photosynthetic performance into the circadian system (Webb *et al.*, 2019) and underscore the importance of PRR7 as an integrator of environmental signals.

## Supplementary data

The following supplementary data are available at *JXB* online.

Fig. S1. Raw and normalized bioluminescence waveforms of data presented in Fig. 1.

Fig. S2. Assessment of circadian rhythms in *xrn* seedlings.

Fig. S3. Assessment of deadenylated RNAs following osmotic stress.

Fig. S4. Relative abundance of polyadenylated RNA following application of osmotic stress.

Fig. S5. Hypocotyl lengths of seedlings in the presence or absence of osmotic stress.

Fig. S6. Normalized bioluminescence waveforms of data presented in Fig. 5.

Table S1. Plant genotypes used in this work.

Table S2. Oligos used for qRT-PCR.

erad274_suppl_supplementary_figures_S1-S6_table_S1-S2

## Data Availability

The genetic lines generated during this study and data in support of its findings are available from the corresponding author on request.
