# Supplementary material for: Inhibition of RNA degradation integrates the metabolic signals induced by osmotic stress into the Arabidopsis circadian system
Source: J Exp Bot. 2023 Jul 15;74(18):5805–19. doi: 10.1093/jxb/erad274 (PMC10540740; doi:10.1093/jxb/erad274)
Supplement: erad274_suppl_supplementary_figures_S1-S6_table_S1-S2 [file erad274_suppl_supplementary_figures_s1-s6_table_s1-s2.pdf]

Supplemental Figure 1

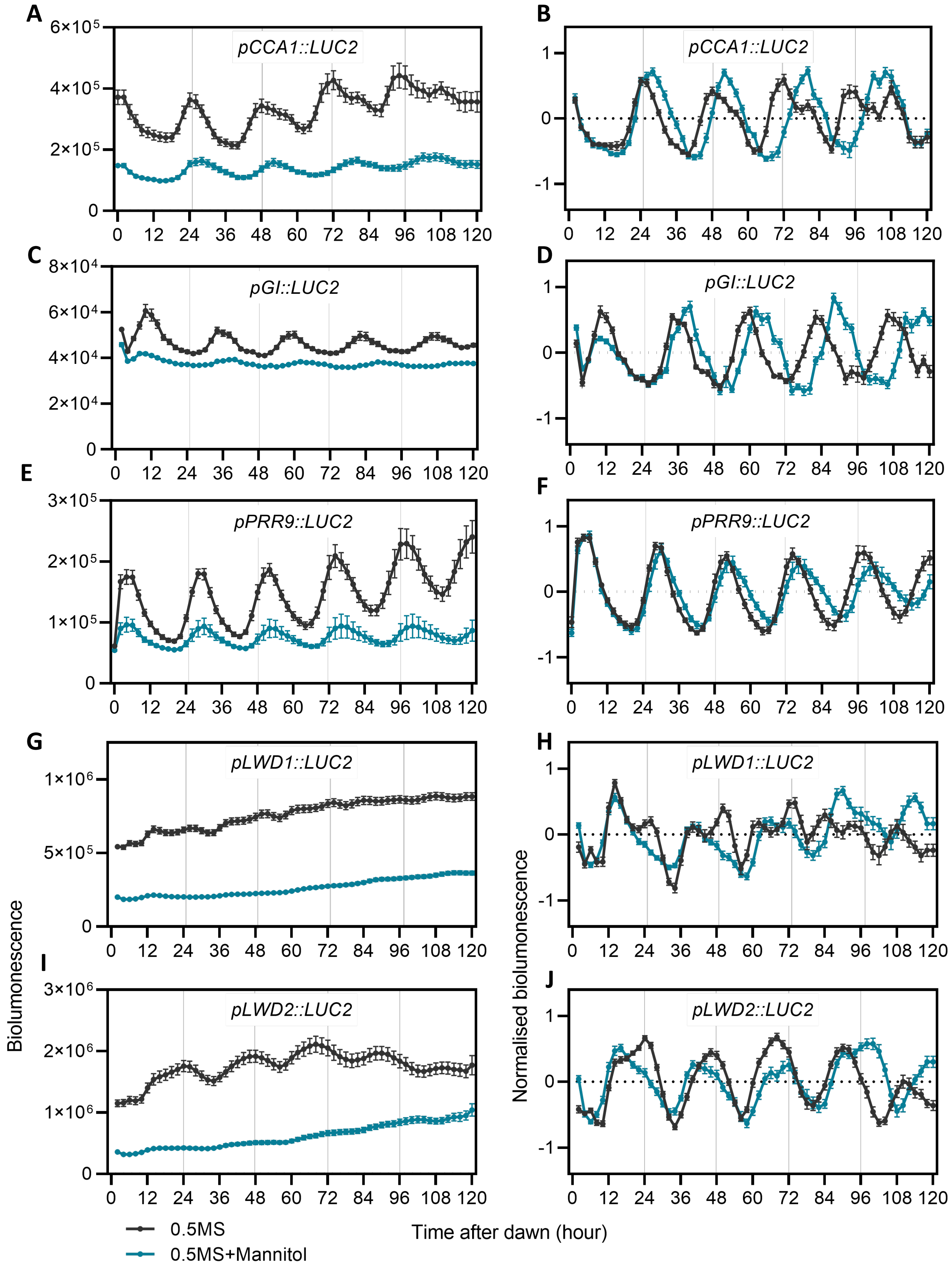

Supplemental Figure 1. Raw and Normalised bioluminescence waveforms of data presented in Figure 1; (A-B) *pCCA1::LUC2*, (**C-D**) *pGI::LUC2* , (**E-F**) *pPRR9::LUC2*, (**G-H**) *pLWD1::LUC2*, (**I-J**) *pLWD2::LUC2*. Data were normalized using BioDare2 (Moore *et* al. 2014). Mean ± s. e. m. are shown.

Supplemental Figure 2

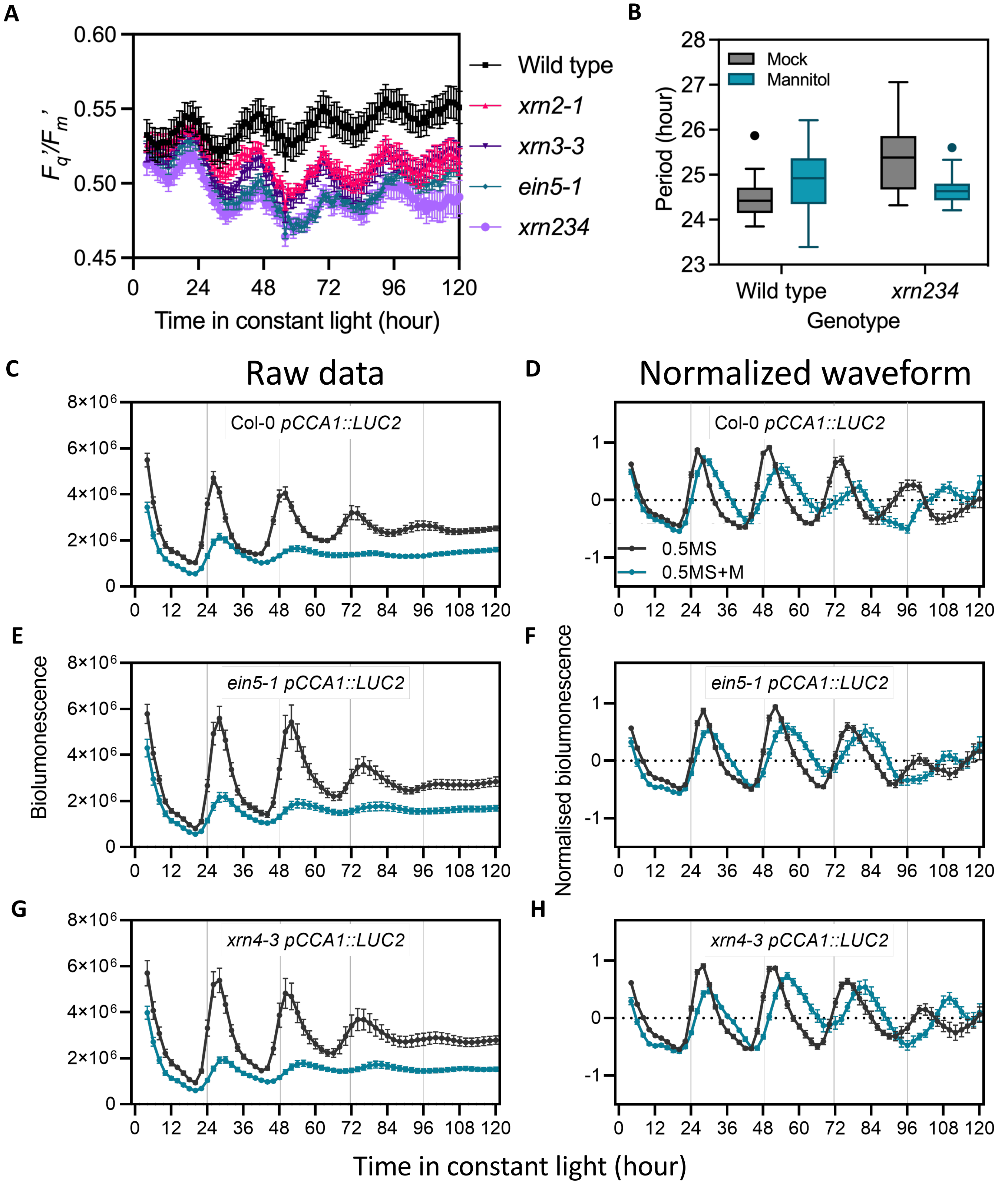

Supplemental Figure 2. Assessment of circadian rhythms in *xrn* seedlings. (A) Circadian rhythms of chlorophyll fluorescence under constant light. Period estimates calculated from these data are shown in Figure 2B. **(B)** Circadian free-running period of Col-0 (wildtype) and *xrn2-1 xrn3-3 xrn4-3* (*xrn234*) seedlings were assessed using chlorophyll fluorescence. Plants were grown on 0.5MS media for 12 d prior to transfer to 200mM mannitol and the commencement of imaging **(C-H)** Raw (C,E,G) and normalized (D, F, H) bioluminescence waveforms data of *pCCA1::LUC2* presented in Figure 2C-F. (C-D) Col-0 (wildtype), (E-F) *ein5-1*, and (G-H) *xrn4-3* background. Data were normalized using BioDare2 (Moore *et* al. 2014). Mean ± s. e. m. are shown. Waveforms presented are representative of three independent experiments.

Supplemental Figure 3

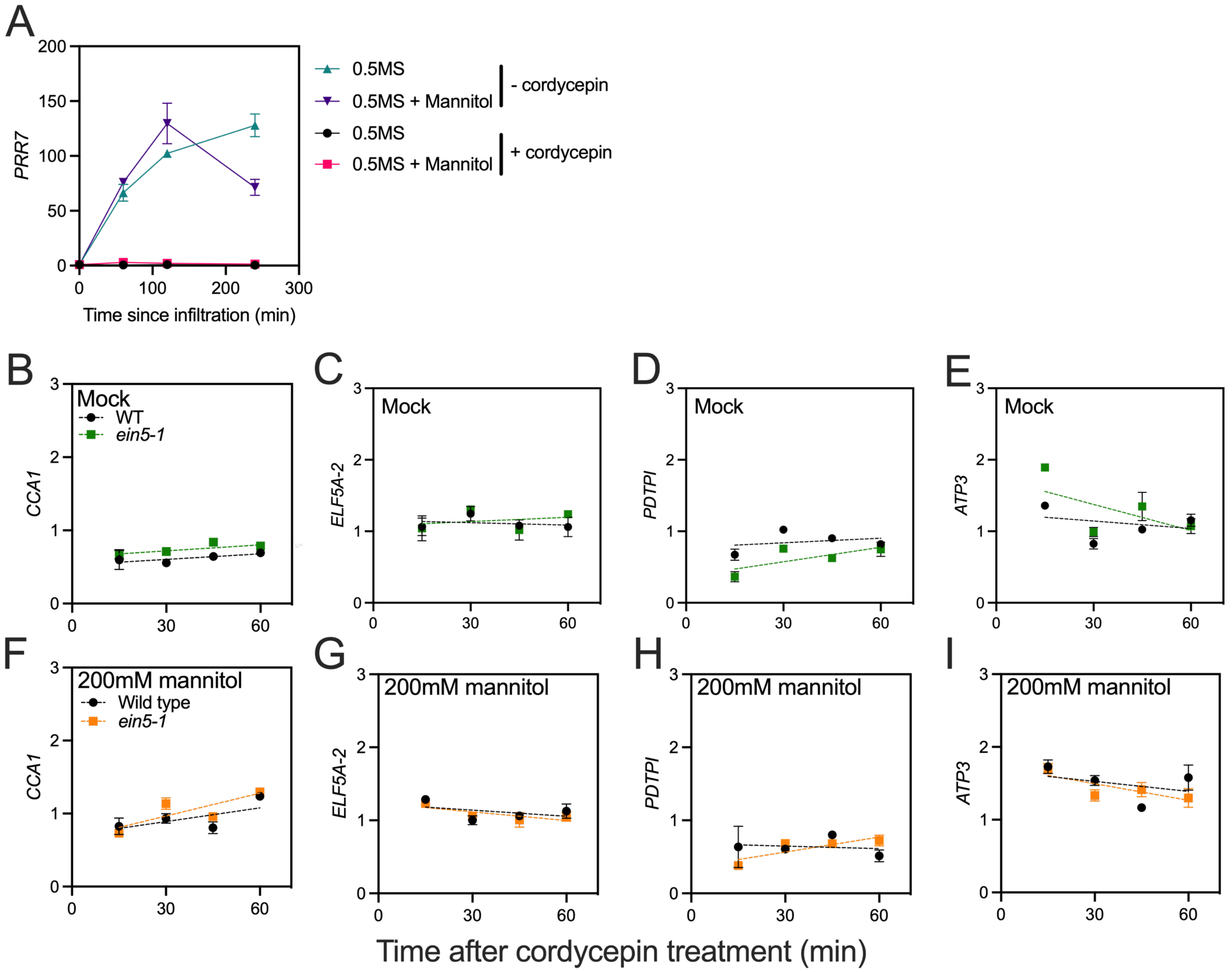

Supplemental Figure 3. Assessment of deadenylated RNAs following osmotic stress. (A) Accumulation of *PRR7* transcript in seedlings following the application of cordycepin. Seedlings were grown on 0.5MS for 6 days prior to transfer to 200 mM mannitol or a mock control media. Sampling and application of 0.5 mM cordycepin was completed at ZT4 on day 7; 28 hours after application of osmotic stress. Data are presented alongside a negative control treated identically, with the exception of cordycepin addition**. (B)** Accumulation of deadenylated *CCA1* relative to control transcripts *ELF5A-2, PDTPI,* and *ATP3.* **(C)** Accumulation of deadenylated *ELF5A-2* relative to *ATP3* and *PDTPI.* **(D)** Accumulation of deadenylated *ATP3* relative to *ELF5A-2* and *PDTPI.* **(E)** Accumulation of deadenylated *PDTPI* relative to *ELF5A-2* and *ATP3.* **(F)** Accumulation of deadenylated *CCA1* relative to control transcripts *ELF5A-2, PDTPI,* and *ATP3* in the presence of osmotic stress. **(G)** Accumulation of deadenylated *ELF5A-2* relative to *ATP3* and *PDTPI* in the presence of osmotic stress. **(H)** Accumulation of deadenylated *ATP3* relative to *ELF5A-2* and *PDTPI* in the presence of osmotic stress. **(I)** Accumulation of deadenylated *PDTPI* relative to *ELF5A-2* and *ATP3* in the presence of osmotic stress. Seedlings were grown on 0.5MS for 6 days prior to transfer to either a mock control or media containing 200 mM mannitol. Sampling and application of 0.5 mM cordycepin was completed at ZT4 on day 7; 28 hours after transfer to experimental media. Data are the mean of at least three independent experiments, n>10. Error bars indicate s. e. m.

Supplemental Figure 4

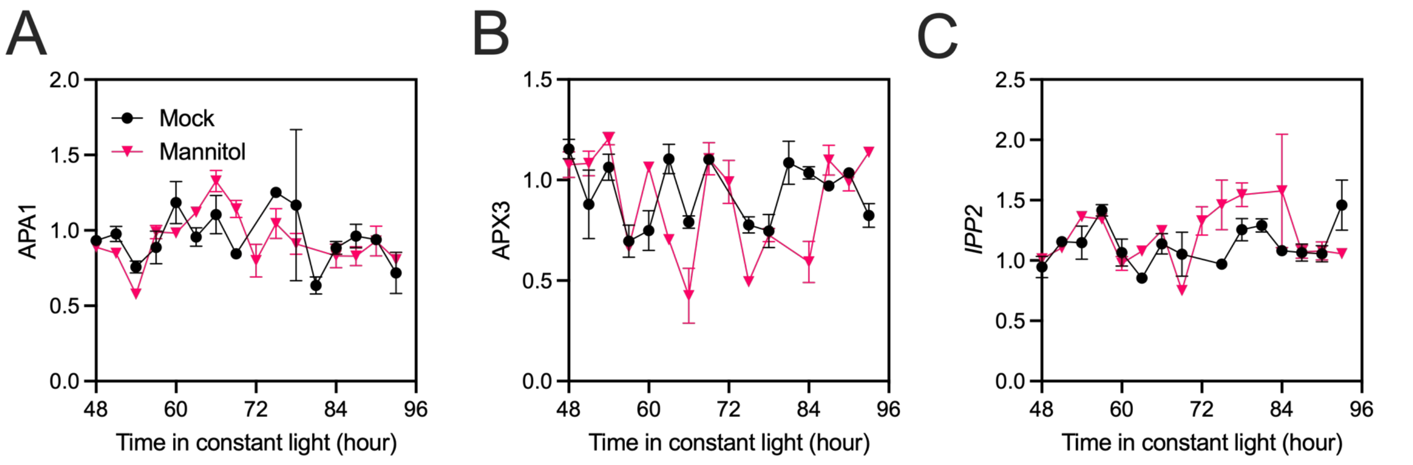

Supplemental Figure 4. Relative abundance of polyadenylated RNA following application of osmotic stress. Seedlings were grown on 0.5 MS media in 12:12 light:dark cycles for 5 days before transfer to 0.5MS in the presence or absence of 200mM mannitol. Seedlings were returned to entraining conditions for 24 hours prior to transfer to continuous white light (60 µmol m^{-2} s^{-1}). Fold-change in *APA1* (A), *APX3* (B), and *IPP2* (C) is presented relative to the other two circadian reference genes listed in Supplemental Table 1. cDNA was synthesized using either an Oligo dT primer. Data are representative of at least three independent experiments (n > 10). Error bars indicate s. e. m.

Supplemental Figure 5

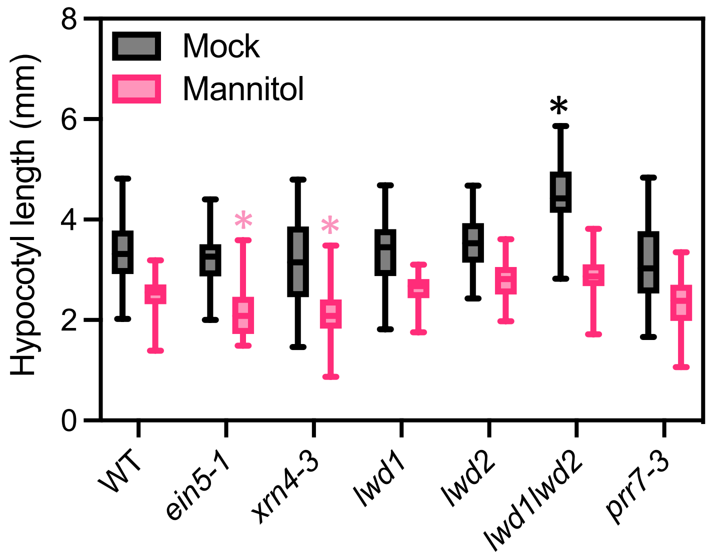

Supplemental Figure 5. Hypocotyl lengths of seedlings in the presence or absence of osmotic stress. Seedlings were germinated on 0.5x MS media for three days prior to transfer to either 200 mM mannitol or a mock control. Seedlings were measured three days after transfer (six days after germination). All genotypes tested had significantly shorter hypocotyls following the application of 200 mM mannitol ($p<0.001$; Šídák's multiple comparisons test). Asterisks indicate significant differences from wild type seedlings in either mock (black asterisks) or stressed conditions (pink asterisks) using Dunnett's multiple comparisons test ($p<0.05$).

Supplemental Figure 6

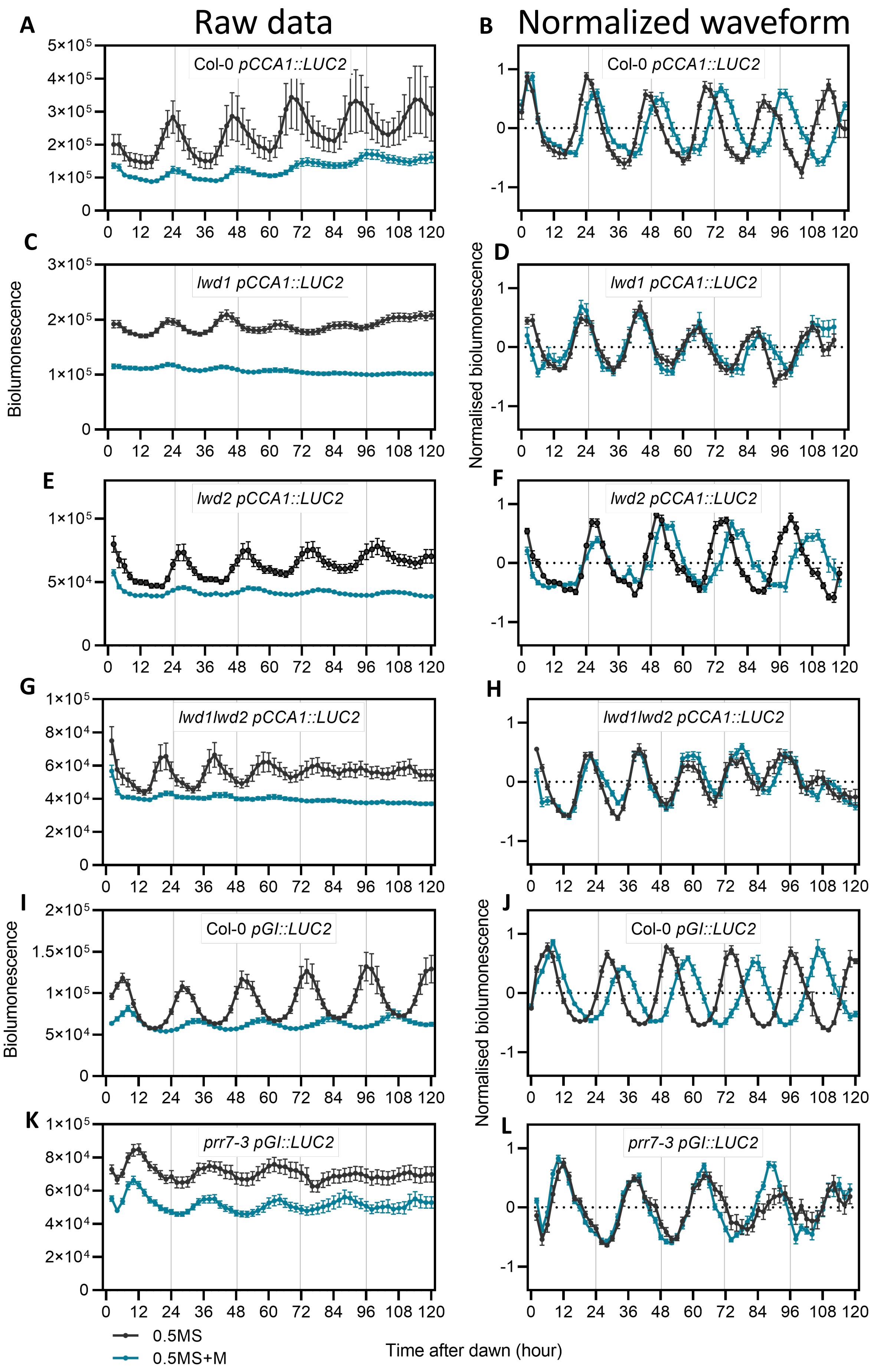

Supplemental Figure 6. Raw and Normalised bioluminescence waveforms of data presented in Figure 5. (**A-D**) *pCCA1::LUC2* reporter in (A-B) Col-0 (wildtype), (C-D) *lwd1*, (E-F) *lwd2*, and (G-H) *lwd1lwd2* background. (**I-L**) *pGI::LUC2* reporter in (**I-J**) Col-0 (wildtype) and (**K-L**) *prr7-3* background. Data were normalized using BioDare2 (Moore *et* al. 2014). Mean ± s. e. m. are shown. Waveform data are representative of three independent experiments.

Supplemental Table 1

Genotype	Target gene/reporter	Description
xrn2-1	*XRN2*	Gy *et al.* 2007
xrn3-3	*XRN3*	Gy *et al.*2007
fry1-6	*FRY1/SAL1*	SALK_020882, Litthauer *et al.* 2018
Col-0	*GI::LUC*	Locke *et al*. 2006
prr7-3	*GI::LUC*	Greenwood *et al.* 2019
Col-0	*CCA1::LUC2*	Jones *et al*. 2015
xrn4-3	*CCA1::LUC*	SALK_014209, Potuschak *et al.* 2006, this study
ein5-1	*CCA1::LUC*	Olmedo *et al*. 2006, this study
lwd1	*CCA1::LUC*	Airoldi *et al.* 2019
lwd2	*CCA1::LUC*	Airoldi *et al.* 2019
lwd1lwd2	*CCA1::LUC*	Airoldi *et al.* 2019
Col-0	*PRR9::LUC*	Locke *et al*. 2006
Col-0	*LWD1::LUC*	Wu *et al.* 2008
Col-0	*LWD2::LUC*	Wu *et al.* 2008

Supplemental Table 1. Plant genotypes used in this work

Supplemental Table 2

GENE	AGI	Experiment	Forward 5'-3'	Reverse 3'-5'
TOC1	AT5G61380	Circadian timecourse	AATAGTAATCCAGCGCAATTTTCTTC	CTTCAATCTACTTTTCTTCGGTGCT
CCA1	AT2G46830	Circadian timecourse	CAGCTCCAATATAACCGATCCAT	CAATTCGACCCTCGTCAGACA
PRR7	AT5G02810	Circadian timecourse and RNA degradation	GAATGTGCTGAGGCGTTCAGA	GGCTGGATTATACCTTGAGAAAGC
LWD1	AT1G12910	Circadian timecourse and RNA degradation	GACCCTATTCTAGCTTACACTGC	ACCCTGAGAATTTGCAGCTTAGT
LWD2	AT3G26640	Circadian timecourse and RNA degradation	CCATTAGAAGAAGAAGACGAAGC	CATTTTGGATTTGATCGCTGCT
APX3	AT4G35000	Reference for circadian timecourses	GCCGTGAGCTCCGTTCTCT	TCGTGCCATGCCAATCG
IPP2	AT3G02780	Reference for circadian timecourses	GTATGAGTTGCTTCTGGAGCAAAG	GAGGATGGCTGCAACAAGTGT
APA1	AT1G11910	Reference for circadian timecourses	CTCCAGAAGAGTATGTTCTGAAAG	TCCCAAGATCCAGAGAGGTC
ATP3	At2g33040	Reference for RNA degradation assays	GAGGGTGAGACGGTCGAG	GTTCCAATTCTCTTGTGTGATGTTC
ELF5A-2	AT1G26630	Reference for RNA degradation assays	ATGGCTTCGTGAGCCTTCTC	CATGACAGACACCACAATATCCTTTC
PDTPI	AT2G21170	Reference for RNA degradation assays	CTGTTCCACGCTGATTTCAC	GTTGTTGTGTCACCTCCATTTG

Supplemental Table 2. Oligos used for qRT-PCR.
